# Genus-Wide Comparative Genomics Analysis of *Neisseria* to Identify New Genes Associated with Pathogenicity and Niche Adaptation of *Neisseria* Pathogens

**DOI:** 10.1155/2019/6015730

**Published:** 2019-01-15

**Authors:** Qun-Feng Lu, De-Min Cao, Li-Li Su, Song-Bo Li, Guang-Bin Ye, Xiao-Ying Zhu, Ju-Ping Wang

**Affiliations:** ^1^School of Medical Laboratory Sciences, Youjiang Medical University for Nationalities, Baise, Guangxi 533000, China; ^2^Center for Scientific Research, Youjiang Medical University for Nationalities, Baise, Guangxi 533000, China; ^3^School of Basic Medical Sciences, Youjiang Medical University for Nationalities, Baise, Guangxi 533000, China

## Abstract

*N. gonorrhoeae* and *N. meningitidis*, the only two human pathogens of *Neisseria*, are closely related species. But the niches they survived in and their pathogenic characteristics are distinctly different. However, the genetic basis of these differences has not yet been fully elucidated. In this study, comparative genomics analysis was performed based on 15 *N. gonorrhoeae*, 75 *N. meningitidis*, and 7 nonpathogenic *Neisseria* genomes. Core-pangenome analysis found 1111 conserved gene families among them, and each of these species groups had opening pangenome. We found that 452, 78, and 319 gene families were unique in *N. gonorrhoeae*, *N. meningitidis*, and both of them, respectively. Those unique gene families were regarded as candidates that related to their pathogenicity and niche adaptation. The relationships among them have been partly verified by functional annotation analysis. But at least one-third genes for each gene set have not found the certain functional information. Simple sequence repeat (SSR), the basis of gene phase variation, was found abundant in the membrane or related genes of each unique gene set, which may facilitate their adaptation to variable host environments. Protein-protein interaction (PPI) analysis found at least five distinct PPI clusters in *N. gonorrhoeae* and four in *N. meningitides*, and 167 and 52 proteins with unknown function were contained within them, respectively.

## 1. Introduction

The *Neisseria* species are a group of Gram-negative, oxidase-positive, *β*-proteobacteria organisms within the family Neisseriaceae. They typically appear in pairs with the adjacent sides flattened and occasionally monococcus or tetrads and grow best at 37°C in the animal body or media [1, 2]. Up to now, at least 30 species of *Neisseria* have been identified (http://www.bacterio.net/neisseria.html). The majority of *Neisseria* species were found primarily on mucosal and dental surfaces in warm-blooded animals as harmless commensals, including *N. lactamica*, *N. elongata*, and *N. mucosa* [2–4]. However, two of them are globally significant pathogens: *N. meningitidis* and *N. gonorrhoeae*.

The *N. meningitides*, a causative agent of meningitis, normally colonize in the upper respiratory tract. It is carried by more than 10% young adults without causing diseases [5]. However, for children under the age of 5 years or adults older than 65 years, it can cause meningococcal disease, which is a life-threatening illness and leads to about 10% case fatality [5, 6] and devastating sequelae, such as deafness and loss of limbs, among survivors. Its serotype distribution varies pronouncedly throughout the world. Six out of thirteen identified capsular types of *N. meningitidis*, including A, B, C, W, X, and Y, account for most disease cases worldwide [7]. The multiple subtypes have hindered the development of vaccines to provide broad-spectrum protection from meningococcal disease [8]. *N. gonorrhoeae* is an obligate human pathogen. It typically causes mucosal infection of the urogenital tract, rectum, pharynx, or eye, even disseminated infections [9, 10]. Untreated *N. gonorrhoeae* infections can cause serious sequelae, such as infertility, urogenital tract abscesses, and adverse pregnancy outcomes, which significantly degrade the quality of life [10]. There are 106.1 million cases of *N. gonorrhoeae* per year in the world [11]. In recent years, the number of cases of gonorrhea has risen significantly. But there is still no effective vaccine to prevent gonorrhea until now [12]. And worse yet, the multidrug-resistant *N. gonorrhoeae* strains have been found widespread emergence [12]. Thus, it is significant to thoroughly understand the adaptive and pathogenic mechanism of *Neisseria* pathogens.

Previous studies found that these two *Neisseria* pathogens shared plenty of virulence genes [13]. In recent years, much work has been done to explore their key factors of virulence, interaction with host cells, mechanism of immune escape, and so forth. For example, type IV pili, encoded by genes *pilC*, *pilD*, *pilE*, *pilS*, etc., is required for initial attachment, twitching motility and competence for natural transformation and autoagglutination [14, 15]. *fHbp* and *nspA*, two immune modulators, can bind to complement factor H to inhibit host immune defenses [16, 17]. With the development of new sequence technologies, enormous genomes of *Neisseria* strains have been sequenced, which makes our understanding of genetic basis of biological characters and biochemical mechanisms more systemic and all-round. Based on comparative genomics of 17 *Neisseria* strains, Marri et al. found that widespread virulence genes exchanged among them and commensal *Neisseria* served as reservoirs of virulence genes [18]. Phase variation was found very prevalent in *Neisseria* pathogens and plays an important role in their niche adaptation and virulence [19, 20].

Although *Neisseria meningitides* and *Neisseria gonorrhoeae* are closely related, the niches they survived in and pathogenic characteristics are distinctly different. The genetic background of these differences has not yet been fully defined. In addition, previous studies focus on a limited number of genes or genomes. There remains a need for a comprehensive picture of similarities and differences of their genome composition to have a better understanding of the genetic basis of their phenotypic features.

In this study, we performed *Neisseria* genus-wide comparative genomics analysis based on all the *Neisseria* complete genomes that are available on public databases. We intended to identify genes that could underlie the apparent differences of specialized niche and pathogenic characteristics of *N. meningitidis* and *N. gonorrhoeae*. Moreover, the genus-wide comparative genomics can give us an overall and profound understanding of genome structure and the evolutional relationships of all the sequenced *Neisseria* species.

## 2. Materials and Methods

### 2.1. Data Retrieval and Genome Management

In this study, the GenBank (.gbk) files of *Neisseria* species with complete genome were retrieved from the National Center for Biotechnology Information (NCBI) genome database (https://www.ncbi.nlm.nih.gov/genome), including 15 *N. gonorrhoeae* strains and 85 *N. meningitidis* strains. Because of few genomes sequenced for nonpathogenic *Neisseria* strains (NPNS), 7 genomes which have at most 10 scaffolds were retrieved. In order to keep the consistence of raw data, the sequences of chromosome, plasmids, and scaffolds for each strain were pasted together into a pseudochromosome by sequence “NNNNNCATTCCATTCATTAATTAATTAATGAATGAATGNNNNN,” which does not affect the genome structure annotation results [21].

To avoid the contradiction that comes from using different annotation pipelines in different research projects, uniform reannotation pipeline was utilized to each genome. In particular, Glimmer v3.02 [22] was used to predict open reading frames (ORFs). The program RNAmmer v1.2 [23] and tRNAscan-SE v1.4 [24] were used to predict ribosomal RNA (rRNA) and transfer RNA (tRNA) genes, respectively.

SSRs of each genome were identified by tandem repeats finder v4.09 [25]. In order to adapt the criteria as the previous study [20, 26], parameters were set as follows: match weight = 4, mismatch penalty = 20, indel penalty = 20, match probability = 80, indel probability = 10, min score to report = 24, and max period size to report = 10. The high penalty values ensured that there were without any mismatches within the repeats and, at the same time, the repeats in which a unit length is greater than 10 bases were discarded.

The candidate contingency loci were identified according to the following criteria. The repeats must be located within the region of an ORF or at most 100 bp upstream of the ORF. Single-base homopolymers must have a length of greater than 6 bp, and dinucleotide repeats must consist of at least 3 repeats. The other repeats with a maximum unit of 10 bases must consist of at least 2 repeats.

### 2.2. Protein Cluster Analysis and Gene Families

All the predicted genes for each *Neisseria* strain were translated into proteomes. Homologous proteins were searched by all-against-all BLASTP comparisons, which means all the proteins existing in one genome against themselves or all the proteins in other genomes [27]. Those pairwise proteins which meet the threshold (identity > 50%, query coverage > 50%, and e-value ≤ 1*e* − 05) were used for further analysis. Then, the Markov clustering algorithm (MCL) [28, 29] was implemented to cluster these blast results. The percentage of homologous proteome for any two genomes was calculated as follows:

(1) $$\begin{array}{c} P_{s}=\frac{N_{s}}{\left(N_{a}+N_{b}\right)}, \end{array}$$

where *P*_s_ is the percentage of shared proteome, *N*_s_ is the number of shared proteins, *N*_*a*_ is the number of one strain protein, and *N*_*b*_ is the number of another strain protein.

The comparison results were displayed in a heat map, which showed the percentage of shared proteome between or within the strain by using the gradation of color.

### 2.3. Phylogenetic Analysis

To investigate the phylogenetic relationship of the 107 *Neisseria* strains, 16s rRNA was used to construct the phylogenetic tree. *Staphylococcus aureus* and *Streptococcus dysgalactiae* were used as outgroups. The multiple sequence alignment was performed by MAFFT v7.123b [30]. Then, the evolutionary history was inferred by the neighbour joining (NJ) [31] method, and the analysis was conducted in MEGA v7 [32] with 1000 bootstrap replications.

To assess the reliability and consistency of the 16s rRNA tree, a genome-scale approach was used to construct the phylogenetic tree [33]. All the single-copy genes were extracted and aligned using MAFFT v7.123b. Then, the results were concatenated for each strain with uniform order. Gblocks v0.91b [34] was used to eliminate poorly aligned positions and divergent regions. Maximum likelihood (ML) and 100 times bootstrap resampling approach were used to compute the phylogenetic tree using RAxML version 8.2.8 [35]. The final tree was visualized by MEGA.

### 2.4. Estimation of Core and Pangenome Size

As follows, two mathematic models [21, 36] were employed to simulate relations between core/pangenome size and genome number, respectively.

Fitting for the pangenome profile model is as follows:

(2) $$\begin{array}{c} y=A_{1}x^{B_{1}}+C_{1}, \end{array}$$

where *y* is the pangenome size, *x* is the genome number, and *A*_1_, *B*_1_, and *C*_1_ are the fitting parameters.

Fitting for the core genome profile model is as follows:

(3) $$\begin{array}{c} y=A_{2}e^{B_{2}x}+C_{2}, \end{array}$$

where *y* is the core genome size, *x* is the genome number, and *A*_2_, *B*_2_, and *C*_2_ are the fitting parameters.

Four genome groups, including all of the tested 107 *Neisseria* strains (ATNS), 85 *N. meningitidis* strains (NMS), 15 *N. gonorrhoeae* (NGS), and 7 nonpathogenic *Neisseria* strains (NPNS), were analyzed and visualized by R-3.2.5 (https://www.r-project.org/), respectively. Specifically, for each group, 100 random permutation lists of all the genomes were generated. Subsequently, we calculated the changes of core/pangenome size at each time a new genome added for each list. Finally, the median values of all counts were used to curve fitting.

### 2.5. Functional Categorization of the Core and Dispensable Genomes

As described in Section 2.4, the dataset was combined into four groups. For core and dispensable genomes of each group, functional annotation and classification were performed using the BLASTP program against Clusters of Orthologous Group (COG, 2014 update, https://www.ncbi.nlm.nih.gov/COG/) database [37], respectively. The function classification results were shown in a bar chart.

### 2.6. Unique Gene Analyses of *N. meningitidis* and *N. gonorrhoeae*

The unique genes for each genome group were identified, based on the protein cluster analysis. The gene families shared by NGS, NMS, and NPNS groups and their combinations were detected by a python script. Then, the statistic results were plotted with a Venn diagram using VennDiagram v1.6.0 [38] in R. The operon predictions of *N. meningitidis* MC58 and *N. gonorrhoeae* 32867 were performed using database for prokaryotic operons (DOOR) v2.0 [39]. Then, function annotations of these genes were performed based on consecutive comparisons against public protein databases as follows: UniProt/Siwss-Prot [40], virulence factor database (VFDB) [41], InterProScan v7 [42], and NCBI nonredundant (NR) protein database [43]. Furthermore, Clusters of Orthologous Groups (COGs, 2014 update) [44] and evolutionary genealogy of genes: nonsupervised orthologous groups (eggNOG) v4.5.1 [45] were used to classify orthologous groups. Finally, all the results above were manually integrated into consolidated results.

### 2.7. SSR Locus Analysis of the Unique Genes for *Neisseria* Pathogens

The distribution of functional category and SSR loci of unique genes for *Neisseria* pathogens were analyzed to assess whether phase-variable genes that code for certain functions were more frequent than expected by chance. The statistic of SSR for each gene set was based on all the corresponding genome sets, and the results were presented as the mean ± standard error.

### 2.8. Protein-Protein Interaction Network Analysis of Unique Protein Sets

To better understand the role of unique protein sets of *Neisseria* pathogens in their niche adaptability and pathogenicity, PPI network analysis was performed using Search Tool for the Retrieval of Interacting Genes/Proteins (STRING v10.5, https://string-db.org/). Then, the PPI results were visualized by Cytoscape 3.6.1 [46].

## 3. Results and Discussion

### 3.1. Genome Statistics and General Features

There are 820 *N. meningitidis* and 434 *N. gonorrhoeae* genome records in NCBI genome database. Additionally, some nonpathogenic *Neisseria* species, such as *N. lactamica*, *N. elongata*, *N. mucosa*, *N. weaveri*, and *N. zoodegmatis*, were sequenced [47–49]. In this study, total of 107 genomes of *Neisseria* strain were used, including 85 *N. meningitidis*, 15 *N. gonorrhoeae* complete genomes, and 7 NPNS, of which six have complete genomes and *N. mucosa* C102 has seven scaffolds (Table 1). Only three *N. gonorrhoeae* strains have plasmid. The average genome size of 107 strains is 2,201,350 bp, ranging from 2,139,957 (*N. meningitidis* LNP21362) to 2,552,522 (*N. zoodegmatis* NCTC12230). The average GC content of all the genomes is 51.76% (*N. gonorrhoeae*: 52.43%, *N. meningitidis*: 51.68%, and NPNS: 51.49%), ranging from 49% (*N. weaveri* NCTC13585) to 54.26% (*N. elongata* ATCC 29315). The average number of open reading frame (ORF) is 2396, ranging from 2132 (*N. mucosa* C102) to 2668 (*N. zoodegmatis* NCTC12230).

According to a survey of some biological characters of *Neisseria* species, they exhibited far more diverse and widespread than previously recognized. For example, members in this genus have a spectrum of morphologies, including bacillus [50, 51], coccobacillus [52], and diplococcus [53], which exists in few bacteria genera. Furthermore, besides humans, some of the *Neisseria* species have been isolated from wide range of animals, such as dog, chimpanzee, and duck [54–56].

### 3.2. Homologous Proteome Analysis by Pairwise Comparisons

**Figure 1:**
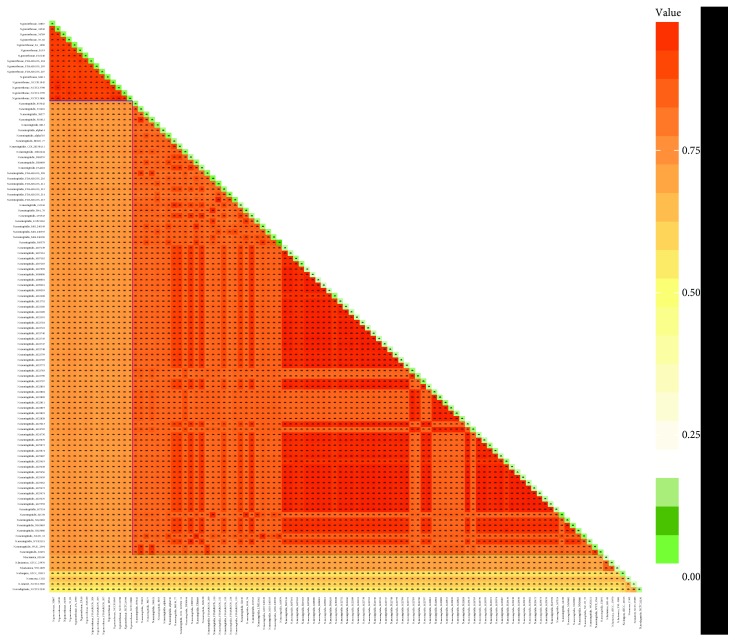
Homologous proteome analysis between different strain proteomes (orthologous) and within a strain proteome (paralogous). The percentages of orthologous and paralogous proteins were represented by orange and green, respectively. The color depth corresponded to the size of homologous proportion. The ratios of homologous were shown in both corresponding boxes and Table S1.

To estimate similarity of *Neisseria* species, the ratios of homologous clusters shared within each strain pair were calculated and visualized (Figure 1). The homologous ratio of different species ranged from 75.21% (*N. gonorrhoeae* NCTC13799 vs. *N. meningitidis* DE10444) to 52.55% (*N. gonorrhoeae* FDAARGOS 205 vs. *N. zoodegmatis* NCTC12230). Within *N. meningitidis* strains, the minimum homologous ratio was as low as 80.38% (*N. meningitidis* FDAARGOS_214 vs. *N. meningitidis* WUE 2594), indicating the high genetic diversity of this species. But for *N. gonorrhoeae* strains, they were generally above 90.75% (*N. gonorrhoeae* FA 1090 vs. *N. gonorrhoeae* FDAARGOS 205). The homologous ratio within genome ranged from 7.19% (*N. meningitidis* M0579) to 1.4% (*N. weaveri* NCTC13585) with average 2.74%, which showed the low redundancy of this genus strain genome composition. For *N. gonorrhoeae*, the homology ratio ranged from 4.49% (*N. gonorrhoeae* 34530) to 3.53% (*N. gonorrhoeae* 32867) with average 4.01%, which was greater than *N. meningitidis*.

### 3.3. Phylogeny of the Genus *Neisseria*

**Figure 2:**
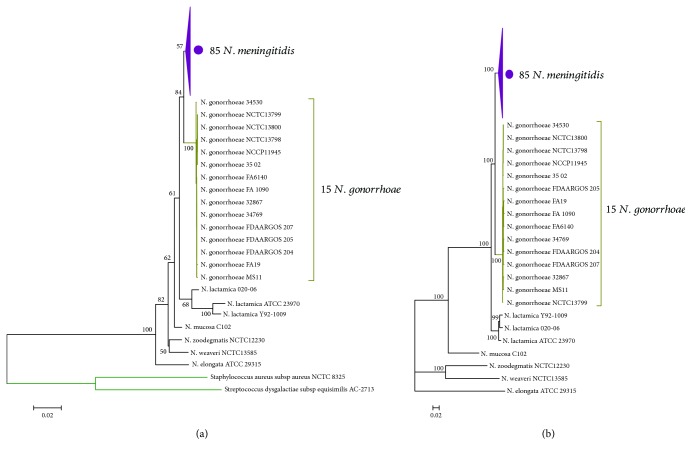
Phylogenetic analysis of *Neisseria* strains included in this study. (a) Phylogenetic tree of 107 *Neisseria* strains constructed by neighbour joining (NJ) approach using 16s rRNA genes with the Kimura 2-parameter substitution model, bootstrapped ×1000 replicates. *Staphylococcus aureus* subsp. *aureus* NCTC 8325 and *Streptococcus dysgalactiae* subsp. *equisimilis* AC-2713 were used as outgroups. Approval values of each major node were indicated. The subtree of *N. meningitidis* was compressed and denoted in purple. (b) Phylogenetic tree constructed by a maximum likelihood (ML) approach using concatenated single-copy gene dataset, bootstrapped ×100 replicates. Approval values of each major node were indicated. The subtree of *N. meningitidis* was compressed and denoted in purple.

It is medically interesting that the *Neisseria* species live in similar habitats but exhibit diverse phenotypes with respect to their interactions with hosts [5, 72, 73]. In order to better understand the evolutionary pattern of *Neisseria* species, a phylogenetic analysis of the genus *Neisseria* was performed, based on 16s rRNA (Figure 2(a)), and conserved amino acid sequences (Figure 2(b)), respectively. The two phylogenetic trees have identical topology, demonstrating the high reliability of the evolutional relationship. In addition, it is obvious that the tree based on single-copy gene dataset has maximum support for a single tree. In line with the previous studies [74, 75], using conserved gene dataset could yield a fully resolved phylogenetic tree with maximum support.

For each tree, each species was clearly distinguished from others. The pathogens, *N. meningitidis* and *N. gonorrhoeae*, were most closely related species and were adjacent to the distal end of the tree, and *N. lactamica* was the closest species to them. Interestingly, from rooted nodes to outer breaches, the morphologies of those species were bacillus (*N. elongata*, *N. weaveri*), coccobacillus (*N. zoodegmatis*), and diplococcus (*N. mucosa*, *N. lactamica*, *N. gonorrhoeae*, and *N. meningitidis*). This may suggest the process of morphological evolution of this genus member.

### 3.4. Core-Pangenome Analysis

**Figure 3:**
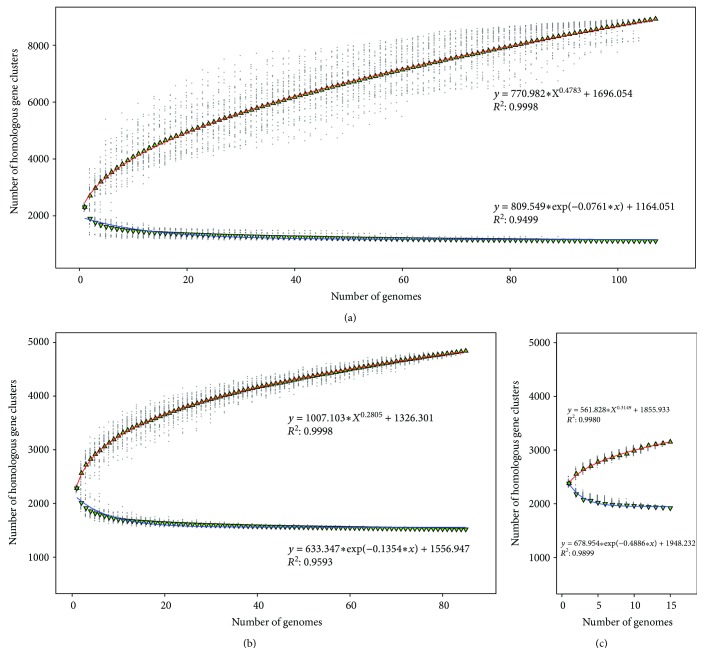
Core and pangenome size evolution. Blue and red curves represent core and pangenome fitting curves for each group, respectively. (a) Core and pangenome of 107 *Neisseria* strain genomes using medians and a power law fit. (b) Core and pangenome of 85 *N. meningitidis* genomes using medians and a power law fit. (c) Core and pangenome of 15 *N. gonorrhoeae* genomes using medians and a power law fit.

In order to estimate the genome polymorphism of *Neisseria* species, the core and pangenome analyses were performed. For all the genomes in the study, the core genome size reached a plateau over 15 additions and finally kept stable at 1111, about half of the average gene content. However, the pangenome size quickly reached 8926 gene clusters, including 3493 singletons, with an average of 62 gene cluster additions for the following each genome addition (Figure 3(a)). Moreover, for 85 *N. meningitidis* genomes, the core and pangenome sizes were 1519 and 4841 gene clusters, respectively (Figure 3(b)). For 15 *N. gonorrhoeae* genomes, the core and pangenome sizes were 1921 and 3153 gene clusters, respectively (Figure 3(c)).

In this study, the power law model was used to describe and predict the trend of *Neisseria* pangenome. The exponent size reflects the characters of pangenome. If it was greater than 0 and less than 1, the pangenome would be open; otherwise, it would be closed [36]. By power law regression of pangenome size, the fitting parameters *B*_1_ kept within the range of 0 ~ 1 (0.4783, 0.2805, and 0.3149 for three groups, respectively), indicating that both *Neisseria* genus and *Neisseria* pathogens (*N. meningitidis* and *N. gonorrhoeae*) had open genome. In order to adapt to a variety of environments, bacteria have to change their genomes, but living in monotone habitats would have smaller pangenome [76]. Most of the *Neisseria* species colonize on the mucosa, which may be the reason that the pangenome size is relatively small compared with other niche diversity species [77, 78].

In contrast to pangenome, the core genome size is relatively stable. However, the core genome sizes of NMS and NGS were greater than ATNS 408 and 810, respectively. This difference showed that there are some unique genes existing in *Neisseria* pathogens, which may be responsible for their pathogenesis characteristics.

### 3.5. Functional Category of Core and Dispensable Genomes

**Figure 4:**
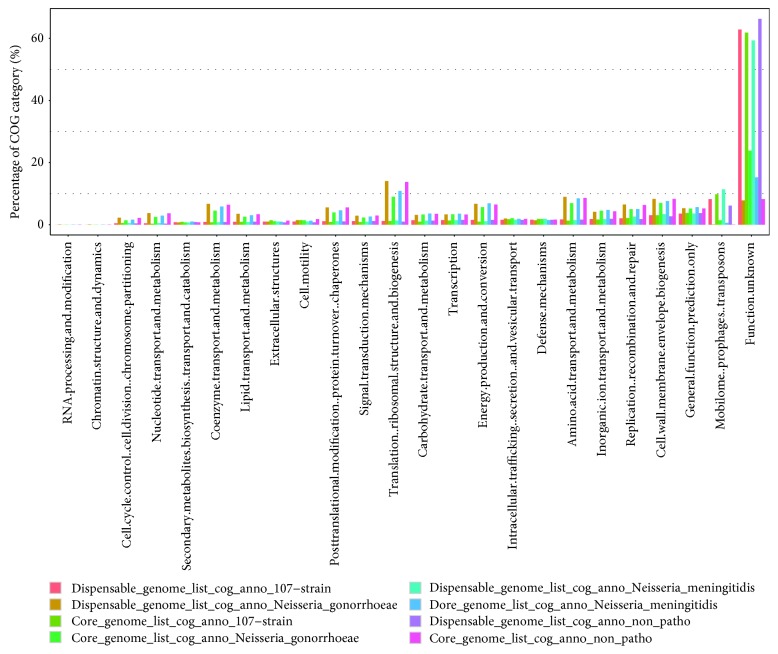
Functional category of core and accessory genome components by COG database. Core and accessory genomes of ATNS, NMS, NGS, and NPNS were showed with different colors, respectively. The genes unassigned to any COG categories were combined into category “function unknown.”

The core genome is always responsible for the basic life process and shared phenotypic characteristics of a group of strains. On the contrary, the dispensable genome, which contributes to the species' own unique characteristics, is probably not essential to their basic life but provides selective advantages, including drug resistance and niche adaptation [79]. The core and dispensable genome sizes of ATNS, NMS, NGS, and NPNS are 1111/7815, 1517/4795, 1921/3786, and 1176/6598, respectively. In the present study, for the core and dispensable genomes of each strain group, functional category was performed using the COG database and divided into 24 subcategories, respectively (Figure 4). The unassigned gene families were merged into the category “function unknown.”

As we expected, most of the core genome proteins for each group play a role of housekeeping. As shown in Figure 4, for the core genome of ATNS, NMS, NGS, and NPNS groups, these function categories that concentrated in were as follows: (1) translation, ribosomal structure, and biogenesis (14.06%, 9.00%, 10.88%, and 13.78%, respectively); (2) amino acid transport and metabolism (8.99%, 6.97%, 8.49%, and 8.61%, respectively); (3) energy production and conversion (6.74%, 5.66%, 6.90%, and 6.50%, respectively); and (4) coenzyme transport and metabolism (6.74%, 4.55%, 5.86%, and 6.42%, respectively). These percentages were much greater than those of dispensable genome, and those functions are basic for life. On the contrary, for the dispensable genome of each group, mobilome: prophages, transposons (8.23%, 9.82%, 11.40%, and 6.14%, respectively) and replication, recombination, and repair (6.49%, 5.03%, 5.07%, and 6.34%, respectively) were the most plentiful categories.

### 3.6. Identification and Annotation of Unique Genes for Each *Neisseria* Group

**Figure 5:**
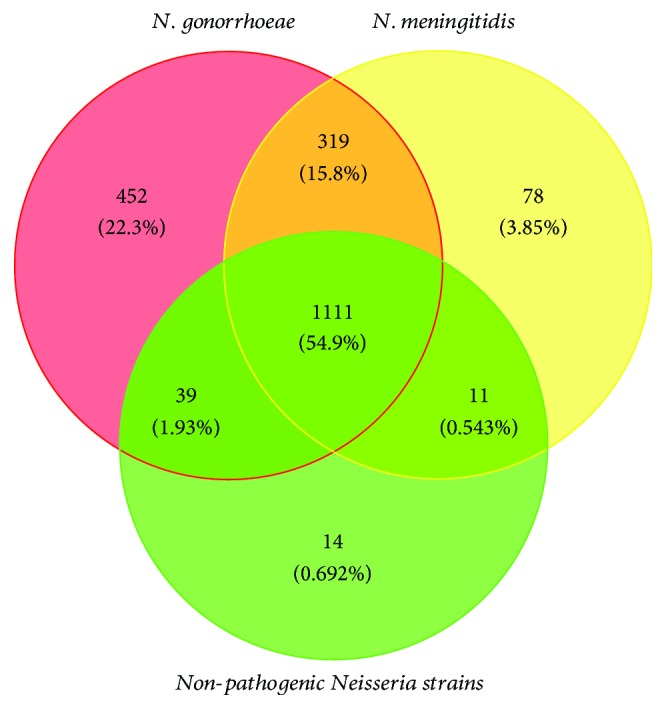
Venn diagram showing the distribution of shared gene families among NMS, NGS, and NPNS. Red, yellow, and green circles represent core genome of *N. gonorrhoeae*, *N. meningitides*, and nonpathogenic *Neisseria* strains, respectively. Their intersections represent the gene families they conserved.

It is a reasonable assumption that unique genome contents of an organism are related directly to its unique phenotypes, which lead to the ability to adapt to unique and complicated conditions of its niche [80]. The number of unique genes for each group, including ATNS, NMS, NGS, and NPNS, was investigated and illustrated with a Venn diagram. As indicated in Figure 5, there are 1111 gene families shared by ATNS, which is in line with preceding core genome analysis results. Interestingly, as many as 319 gene families were unique genes for *Neisseria* pathogens (UGNP) but absent in NPNS. Moreover, NMS and NGS shared 11 and 39 gene families only with NPNS, respectively. Furthermore, there were 78 unique genes for NMS (UGNMS) and 452 unique genes for NGS (UGNGS). These unique genes of *Neisseria* pathogens may be the key factors which are related with their niche adaptability and pathogenicity. To some extent, the sample sizes of NGS and NPNS have an impact on reliability of the unique genes. More *Neisseria* samples should be sequenced in the future. In order to comprehend the roles of these unique genes in *Neisseria* pathogens, we investigated the gene functions of UGNP, UGNMS, and UGNGS.

The UGNP genes (Table S2) were enriched in COG categories C: energy production and conversion (average 7.98% for *N. gonorrhoeae*; average 8.24% for *N. meningitidis*, the same order with the following), E: amino acid transport (7.15%; 7.36%), and P: inorganic ion transport and metabolism (6.38%; 6.56%), significantly. Those genes are associated with basic metabolism, and many of them have been proved to be important factors surviving in their niche [81]. For example, Na^+^-transporting NADH:ubiquinone oxidoreductase (Na^+^-NQR, opr_724 for *N. gonorrhoeae* 32867; opr_285 for *N. meningitidis* MC58) was found conservative in plenty of bacteria pathogens such as *Vibrio cholerae* [82], *Klebsiella pneumoniae* [83], and *Yersinia pestis* [84]. This enzyme pumps Na^+^ across the cell membrane to generate a sodium motive force that can be used for solute import, ATP synthesis, and flagella rotation [85]. In *V. cholerae*, it was considered as an important factor to induce virulence factors [82]. Besides, many high-affinity iron uptake systems, which facilitate acquisition of the essential irons in the host, were found unique in *Neisseria* pathogens. ABC transporters, *fbpA* and *fbpB*, transcribed as an operon (opr_122 for *N. gonorrhoeae* 32867; opr_312 for *N. meningitidis* MC58), are necessary for the utilization of iron bound to transferrin or iron chelates [86, 87]. Furthermore, many other UGNP proteins, including Mn^2+^ efflux pump (*MntP*), multidrug resistance translocase (*farB*), and factor H-binding protein (*fHbp*), have been found to play important roles in niche adaptation [16, 88, 89].

For the UGNGS genes (Table S3), COG categories X: mobilome: prophages, transposons (average 6.43%), M: cell wall/membrane/envelope biogenesis (average 4.62%), and P: inorganic ion transport and metabolism (average 3.89%) were enriched. Similarly, for the UGNMS genes (Table S3), COG categories X (average 11.39%), U: intracellular trafficking, secretion, and vesicular transport (average 7.54%), and P (average 4.91%) were enriched. Substantial mobilome suggests that horizontal gene transfer may be widespread and frequent occurrence in *N. gonorrhoeae* genomes, which is beneficial for them to survive in changeable environmental conditions and develop resistance [90, 91]. In addition, most proteins of COG categories M, P, and U are membranes or membrane-associated proteins, such as glycosyltransferase involved in LPS biosynthesis (*lgtB*, *lgtE*: opr_1140 of *N. gonorrhoeae* 32867) and large exoprotein involved in heme utilization or adhesion (opr_252, opr_253, opr_256, opr_257 of *N. meningitidis* MC58), which play vital roles in interaction with the host and environment [92, 93]. What is more, the composition differences between UGNGS and UGNMS may be contributing greatly to their different tissue tropisms and pathogenic characteristics.

As detailed in Tables S2 and S3, a large number of genes that conserved in each species group were clustered into operons and had synergistic effects on its pathogenicity and niche adaptation. However, for each gene set, at least one-third genes (average 31.17% for UGNP, 52.38% for UGNGS, and 35.0% for UGNMS) have not been found the certain annotation information, indicating that the study for *Neisseria* species is not sufficient, and many more studies are still required to be done in the future.

### 3.7. SSR Locus Identification and COG Enrichment Analyses of Unique Genes of *Neisseria* Pathogens

**Figure 6:**
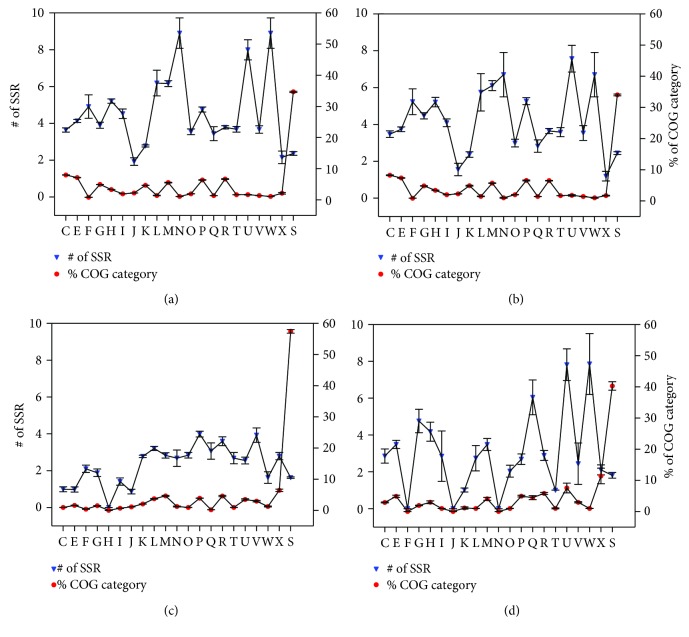
COG enrichment analysis of SSR loci in NMS and NGS unique genes. Subplots (a) and (b) represent the UGNP gene set characteristics based on *Neisseria gonorrhoeae* and *Neisseria meningitidis* datasets, respectively. Subplot (c) represents the UGNGS gene set characteristics based on 15 *Neisseria gonorrhoeae* strains, and subplot (d) represents the UGNMS gene set characteristics based on *Neisseria meningitidis* strains. The red circular markers represent the average percentage of genes that enriched in the COG function categories (C–X) for each genome dataset. The blue inverted triangle markers represent the average number of SSR of each COG category gene for each genome dataset. The error bar represents the standard error of the mean for each gene group. Since the gene is absent from five COG categories (A, B, D, Y, and Z) in presented dataset, they have been omitted from the figures. Besides, the genes unassigned to any COG categories were combined into category S (function unknown). COG abbreviations: C: energy production and conversion; E: amino acid transport and metabolism; F: nucleotide transport and metabolism; G: carbohydrate transport and metabolism; H: coenzyme transport and metabolism; I: lipid transport and metabolism; J: translation, ribosomal structure, and biogenesis; K: transcription; L: replication, recombination, and repair; M: cell wall/membrane/envelope biogenesis; N: cell motility; O: posttranslational modification, protein turnover, and chaperones; P: inorganic ion transport and metabolism; Q: secondary metabolite biosynthesis, transport, and catabolism; R: general function prediction only; T: signal transduction mechanisms; U: intracellular trafficking, secretion, and vesicular transport; V: defense mechanisms; W: extracellular structures; X: mobilome: prophages, transposons; S: function unknown.

In many microbial pathogens, it has been found ubiquitous that SSRs were used in genes, which are mostly involved in host interactions, such as antigenic variation, to generate phase variation or protein sequence diversity, and this has been considered to contribute greatly to their virulence and adaptation [94, 95]. So, we investigated the distribution of SSR loci in each COG category for UGNP, UGNMS, and UGNGS genes (Figure 6), respectively.

In UGNP genes (Figures 6(a) and 6(b)), the average numbers of SSR for about one half COG categories were greater than 4. It is interesting that W: extracellular structures (average 8.9 for *N. gonorrhoeae*; 6.7 for *N. meningitidis*, the same order with the following); U: intracellular trafficking, secretion (8.0; 7.6), and vesicular transport; N: cell motility (8.9; 6.7); and M: cell wall/membrane/envelope biogenesis (6.2; 6.1) were the SSR-enriched genes. Obviously, most of these genes are membrane or related proteins (Table S1), such as type IV pilus proteins (*pilC*, *pilP*, and *pilV*) [96] and type V secretory pathway [97] (as detailed in Table S1), which are associated with virulence, niche adaptation, or other host interactions. Those genes have a high rate of mutation via slipped-strand mispairing at SSR loci during replication, which helps the *Neisseria* pathogens adapt to vastly different environments and evade host immune systems [19, 20, 94]. Additionally, the phase variation of these genes that encode surface-associated antigens is a big challenge to develop clinically efficient vaccine [98].

According to Figures 6(c) (based on UGNGS dataset) and 6(d) (based on UGNMS dataset), the average number of SSR of most COG categories of UGNMS is greater than UGNGS, especially in W: extracellular structures (1.6; 7.9), U: intracellular trafficking, secretion, and vesicular transport (2.6; 7.8), and Q: secondary metabolite biosynthesis, transport, and catabolism (3.1; 6.0). Eleven large exoproteins with average about 7 SSRs per gene (located in operons: opr_252, opr_253, opr_256, opr_257, opr_884, opr_885, and opr_886 of *N. meningitidis* MC58), which are involved in heme utilization or adhesion, were found in UGNMS. For UGNGS, eleven restriction-modification system-associated proteins, which are important to defense against foreign DNA, were identified as SSR-rich genes (average 4.5 SSRs per gene). Besides, phase variation of those DNA methyltransferases alters global DNA methylation patterns, which is associated with the epigenetic regulation of gene expression of multiple proteins that are involved in colonization, infection, and resistance to host defense, to aid *N. gonorrhoeae* adaptation to changing circumstance [99].

### 3.8. Protein-Protein Interaction Network Analyses of Unique Genes

**Figure 7:**
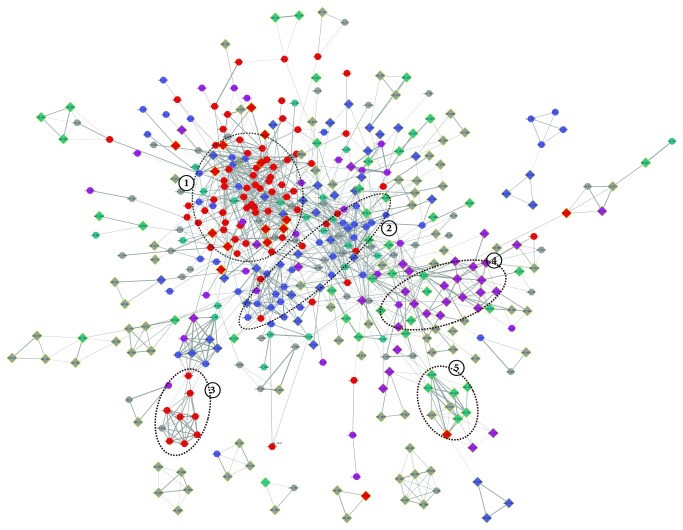
Protein-protein interaction of UGNP and UGNGS. *N. gonorrhoeae* 32867 genome is used as reference. The names of nodes correspond to Table S1. Circular nodes represent the UGNP proteins, and diamond nodes with yellow margin represent the UGNGS proteins. Only the networks with the number of nodes greater than 3 were shown. The network edges represent the protein-protein associations, and their line thickness indicates the confidence of the association between corresponding nodes. The disconnected nodes are hidden in the map. Different colors reflect different protein function categories: red: basic substance transport and metabolism; purple: genetic information processing, including replication, transcription, and translation; blue: cellular processes, including cell wall/membrane/envelope biogenesis and cell motility; green: bacteria-environment interaction, including signal transduction, extracellular structures, and defense mechanism; grey, function unknown.

The unique genes of *N. gonorrhoeae* or *N. meningitidis* which is absent from NPNS were analyzed by STRING to construct the protein-protein interaction (PPI) network map. As showed in Figure 7, 489 proteins were contained in the *N. gonorrhoeae* PPI network map, including 244 UGNP proteins and 245 UGNGS proteins. Obviously, the network map had at least 5 major PPI clusters, and the proteins within them may interact with each other to function properly.

Most proteins of clusters 1 and 2 were UGNP proteins, and functional enrichment analysis indicated that they were involved in basic substance transport/metabolism and cellular processes associated with interacting networks, respectively. Specifically, ABC-type amino acid transport/signal transduction system (orf_439-orf_441, orf_2359-orf_2362), energy production and conversion-associated PPI network (orf_577, orf_778, orf_779, orf_1719, orf_1771, orf_1774, etc.), and so on were contained in cluster 1. They were important enzymes involved in signaling pathways and metabolic processes. In cluster 2, there was a cell wall polysaccharide biosynthesis system (orf_491, orf_1557, orf_2021, orf_2442-orf_2447, orf_2525, etc.), which was involved in immune system evasion, attachment to epithelial tissue, and an important mediator of the proinflammatory response [100]. Besides, the ion recognition and transport system (orf_264, orf_265, orf_331, orf_2462, orf_1513-orf_1515, orf_1585, etc.) have been proved crucial to the survival of *Neisseria* pathogens in vivo [101]. The cluster 3 codes for Na^+^-translocating NADH:ubiquinone oxidoreductase (orf_1633-orf_1638, orf_1640, orf_1641, and orf_1659), which was found widely in pathogenic and conditionally pathogenic bacteria and shown to be important for the induction of virulence factors [82, 102].

However, most proteins of cluster 4 came from UGNGS and they were associated with DNA methylation and repairing (orf_8, orf_10, orf_361, orf_362, orf_431, orf_665, orf_825, orf_1109, orf_2226, etc.). They were related to the fine tuning of gene expression and DNA repair to aid *N. gonorrhoeae* adaptation to changing circumstance [99]. Moreover, cluster 5 was also unique in *N. gonorrhoeae* (orf_1074–orf_1077, orf_1079, orf_476, orf_477, and orf_479). Those genes code for restriction endonucleases. Weyler et al. found restriction endonucleases, which were released by intracellular *Neisseria gonorrhoeae*, damaged human chromosomal DNA, and distorted mitosis [103]. Besides, some other pathogenic-associated clusters were also found in Figure 7 network, such as the nitric oxide metabolic pathway (orf_1612-orf_1620) [104] and Tfp pilus assembly protein (orf_65, orf_129, orf_1665, orf_2249, orf_528, orf_1467, and orf_1757) [105].

For *N. meningitidis*, 252 proteins were contained in the PPI network map (Figure S1), including 203 UGNP proteins and 49 UGNMS proteins. The PPI clusters 1, 2, and 3 in *N. meningitidis* were found to be similar with *N. gonorrhoeae*. Moreover, cluster 4 associated with heme utilization or adhesion (orf_543, orf_545, orf_548, orf_551, orf_551, orf_556, orf_1971, orf_1983, and orf_1984) was found in Figure S1 network.

As analyzed above, plenty of proteins have been identified as crucial determinants in the context of colonization and invasive capability. Those unique proteins in *N. meningitides* and *N. gonorrhoeae* may account for the differences of pathogenicity and their niche adaptation. However, 167 proteins within Figure 7 and 52 proteins within Figure S1 still have no definite functional annotation at present. Their functions should be studied in the future.

## 4. Conclusions

*N. meningitidis* and *N. gonorrhoeae*, the closely related human pathogens with distinct habitat niches and pathogenic features, have been studied deeply. However, the genetic background of these differences has not yet been fully elucidated. In the present investigation, genus-wide comparative genomics analysis of *Neisseria* was performed to identify genes associated with pathogenicity and niche adaptation, based on sequenced genome of NMS, NGS, and NPNS. The core and dispensable genome sizes of ATNS, NMS, NGS, and NPNS are 1111/7815, 1517/4795, 1921/3786, and 1176/6598, respectively. The power law regression analysis of pangenome found that both *Neisseria* genus and each *Neisseria* pathogen have open pangenome (Figure 3). Most of the *Neisseria* species colonize on the mucosa but in various individuals, which may lead to the open pangenome but relatively small size compared with other niche diversity species [77, 78]. Secondly, the number of UGNP, UGNMS, and UGNGS is 319, 78, and 452, respectively. Functional analysis indicated that plenty of them have been proved as ones that playing significant roles in their pathogenicity and niche adaptation. Moreover, SSR locus identification and COG enrichment analysis of those unique genes showed that a large number of host interaction-associated proteins, especially membrane or related ones, were enriched with SSR. These results are in good agreement with previous observations [20]. Finally, the PPI analyses of *N. meningitidis* and *N. gonorrhoeae* unique proteins found that the majority of UGNP proteins were markedly clustered into two clusters (Figure 7, Figure S1). Functional enrichment analysis indicated that they are basic substance transport/metabolism and cellular processes associated with interacting networks, respectively. Some other clusters were also found, such as restriction-modification system, nitric oxide metabolic pathway, and heme utilization or adhesion system. Those proteins unique in *N. meningitides* and *N. gonorrhoeae* may well be vital to the niche adaptation and pathogenicity of the corresponding *Neisseria* species. However, 167 proteins with unknown function of *N. gonorrhoeae* and 52 of *N. meningitidis* exist in PPI analysis maps. They may interact with others and should be investigated in the future. What is more, the methods used in this study could be applied to other species to infer relationships between phenotypes and genotypes.

## Figures and Tables

**Table 1:** Genomic details of *Neisseria* species used in the present study.

Organism	Accession number	Genome size (bp)	GC content (%)	# of scaffolds	# of plasmids	# of ORFs	rRNA	tRNA	Morphology	Natural host	Reference
*Neisseria gonorrhoeae*									Diplococcus	Human	
32867	CP016015	2,218,818	52.41	1	0	2545	12	55			
34530	CP016016	2,228,373	52.35	1	0	2557	12	55			
34769	CP016017	2,220,340	52.43	1	0	2541	12	55			
35_02	CP012028	2,173,235	52.6	1	0	2468	12	55			[57]
FA_1090	AE004969	2,153,922	52.69	1	0	2448	12	55			
FA19	CP012026	2,232,367	52.38	1	0	2573	12	55			[57]
FA6140	CP012027	2,168,698	52.59	1	0	2485	12	55			[57]
FDAARGOS_204	CP020415	2,212,422	52.45	1	0	2541	12	55			
FDAARGOS_205	CP020417	2,278,895	52.26	1	2	2665	12	54			
FDAARGOS_207	CP020419	2,189,343	52.36	1	0	2497	12	55			
MS11	CP003909	2,237,793	52.36	1	1	2591	12	55			
NCCP11945	CP001051	2,236,178	52.36	1	1	2574	12	55			[58]
NCTC13798	NZ_LT906440	2,230,241	52.35	1	0	2551	12	55			
NCTC13799	NZ_LT906437	2,172,222	52.56	1	0	2469	12	55			
NCTC13800	NZ_LT906472	2,226,638	52.36	1	0	2549	12	55			
*Neisseria meningitidis*									Diplococcus	Human, chimpanzee	
53442	CP000381	2,153,416	51.7	1	0	2298	12	59			[59]
331401	CP012694	2,191,116	51.86	1	0	2361	12	59			
38277	CP015886	2,264,278	51.4	1	0	2458	12	60			
510612	CP007524	2,188,020	51.83	1	0	2368	12	59			[60]
8013	FM999788	2,277,550	51.43	1	0	2415	12	59			[61]
alpha14	AM889136	2,145,295	51.95	1	0	2279	12	58			[62]
alpha710	CP001561	2,242,947	51.69	1	0	2406	12	57			[63]
B6116_77	CP007667	2,187,672	51.66	1	0	2386	12	59			[64]
COL-201504-11	CP017257	2195,573	51.65	1	0	2372	12	59			
DE10444	CP012392	2,170,619	51.63	1	0	2279	12	59			
DE8555	CP012393	2,207,932	51.81	1	0	2394	12	59			
DE8669	CP012391	2,230,103	51.42	1	0	2362	12	59			
FAM18	AM421808	2,194,961	51.62	1	0	2324	12	59			[65]
FDAARGOS_209	CP020420	2,181,227	51.89	1	0	2364	12	59			
FDAARGOS_210	CP020421	2,273,677	51.52	1	0	2414	12	59			
FDAARGOS_211	CP020422	2,305,790	51.43	1	0	2469	12	59			
FDAARGOS_212	CP020423	2,244,857	51.5	1	0	2426	12	59			
FDAARGOS_214	CP020401	2,397,439	51.05	1	0	2621	12	59			
FDAARGOS_215	CP020402	2,305,808	51.43	1	0	2473	12	59			
G2136	CP002419	2,184,862	51.68	1	0	2352	12	59			[66]
H44_76	CP002420	2,240,883	51.45	1	0	2379	12	59			[66]
L91543	CP016684	2,173,191	51.72	1	0	2357	12	59			
LNP21362	CP006869	2,139,957	51.82	1	0	2297	12	59			
M01-240149	CP002421	2,223,518	51.44	1	0	2360	12	59			[66]
M01-240355	CP002422	2,287,777	51.5	1	0	2413	12	59			[66]
M04-240196	CP002423	2,250,449	51.4	1	0	2383	12	59			[66]
M0579	CP007668	2324,822	51.37	1	0	2456	12	59			
M07149	CP016650	2,173,513	51.73	1	0	2377	12	59			
M07161	CP016675	2,169,790	51.75	1	0	2385	12	59			
M07162	CP016644	2,193,742	51.67	1	0	2413	12	59			
M07165	CP016880	2,207,174	51.58	1	0	2442	12	59			
M07999	CP016881	2,162,082	51.77	1	0	2361	12	59			
M08000	CP016681	2,162,376	51.77	1	0	2357	12	59			
M08001	CP016652	2,162,199	51.78	1	0	2359	12	59			
M09261	CP016665	2,154,341	51.82	1	0	2351	12	59			
M09293	CP016648	2,161,510	51.78	1	0	2366	12	59			
M10208	CP009422	2,183,230	51.68	1	0	2434	12	59			
M12752	CP016645	2,173,879	51.73	1	0	2370	12	59			
M22160	CP016674	2,177,343	51.73	1	0	2381	12	59			
M22189	CP016649	2,172,699	51.74	1	0	2372	12	59			
M22191	CP016683	2,155,494	51.82	1	0	2337	12	59			
M22718	CP016627	2,173,408	51.73	1	0	2367	12	59			
M22722	CP016663	2,172,888	51.73	1	0	2370	12	59			
M22740	CP016679	2,172,899	51.73	1	0	2366	12	59			
M22745	CP016657	2,163,399	51.77	1	0	2357	12	59			
M22747	CP016882	2,173,015	51.73	1	0	2375	12	59			
M22748	CP016653	2,157,503	51.78	1	0	2359	12	59			
M22759	CP016669	2,168,308	51.74	1	0	2342	12	59			
M22769	CP016656	2,168,495	51.74	1	0	2344	12	59			
M22772	CP016655	2,173,607	51.73	1	0	2366	12	59			
M22783	CP016671	2,180,570	51.64	1	0	2334	12	59			
M22790	CP016883	2,168,169	51.67	1	0	2360	12	59			
M22797	CP016884	2,193,498	51.76	1	0	2406	12	59			
M22801	CP016659	2,172,426	51.73	1	0	2376	12	59			
M22804	CP016660	2,174,791	51.65	1	0	2325	12	59			
M22809	CP016647	2,182,171	51.63	1	0	2332	12	59			
M22811	CP016654	2,185,698	51.62	1	0	2342	12	59			
M22819	CP016646	2,173,686	51.65	1	0	2308	12	59			
M22822	CP016680	2,173,901	51.67	1	0	2334	12	59			
M22828	CP016672	2,172,926	51.67	1	0	2318	12	59			
M23413	CP016662	2,173,723	51.73	1	0	2372	12	59			
M24705	CP016682	2,175,832	51.66	1	0	2327	12	59			
M24730	CP016658	2,175,362	51.74	1	0	2369	12	59			
M25070	CP016664	2,168,001	51.73	1	0	2372	12	59			
M25073	CP016885	2,204,403	51.59	1	0	2415	12	59			
M25074	CP016886	2,211,323	51.56	1	0	2440	12	59			
M25087	CP016670	2,167,991	51.72	1	0	2365	12	59			
M25419	CP016678	2,189,560	51.71	1	0	2373	12	59			
M25438	CP016661	2,171,975	51.74	1	0	2369	12	59			
M25456	CP016677	2,173,106	51.73	1	0	2371	12	59			
M25459	CP016673	2,173,115	51.73	1	0	2376	12	59			
M25462	CP016666	2,174,042	51.73	1	0	2378	12	59			
M25472	CP016668	2,170,146	51.72	1	0	2370	12	59			
M25474	CP016651	2,172,576	51.73	1	0	2364	12	59			
M25476	CP016676	2,168,191	51.73	1	0	2370	12	59			
M27559	CP016667	2,173,745	51.73	1	0	2369	12	59			
M7124	CP009419	2,179,483	51.73	1	0	2372	15	61			
MC58	AE002098	2,272,360	51.53	1	0	2412	12	59			[67]
NM3682	CP009420	2,196,674	51.64	1	0	2388	13	59			
NM3683	CP009421	2,199,215	51.64	1	0	2384	14	61			
NM3686	CP009418	2195,266	51.65	1	0	2365	12	59			
NZ-05_33	CP002424	2,248,966	51.33	1	0	2385	12	59			[66]
WUE2121	CP012394	2,206,847	51.82	1	0	2403	12	59			
WUE_2594	FR774048	2,227,255	51.84	1	0	2425	12	55			[68]
Z2491	AL157959	2,184,406	51.81	1	0	2335	12	58			[69]
*Nonpathogenic Neisseria species*											
*N. lactamica* 020-06	FN995097	2,220,606	52.28	1	0	2372	12	59	Diplococcus	Human	[70]
*N. lactamica* ATCC 23970	KN046803	2,182,033	52.17	1	0	2294	12	57	Diplococcus	Human	
*N. lactamica* Y92-1009	CP019894	2,146,723	52.34	1	0	2298	12	58	Diplococcus	Human	
*N. mucosa* C102	GL635799	2,169,437	49.42	7	0	2132	3	54	Diplococcus	Human, dog, duck, dolphin	
*N. weaveri* NCTC13585	LT571436	2,188,497	49	1	0	2195	12	55	Bacillus	Human, dog	
*N. zoodegmatis* NCTC12230	NZ LT906434	2,552,522	50.94	1	0	2668	12	55	Coccobacillus	Human, dog	
*N. elongata* ATCC 29315	CP007726	2,256,647	54.26	1	0	2464	12	61	Coccobacillus	Human	[71]

Note. Latin name, accession number, genome size, GC content, number of scaffolds, number of plasmids, number of ORFs, rRNA, tRNA, morphology, natural host, and reference are listed.

## Data Availability

The data used to support the findings of this study are available from the corresponding author upon request.

## Abbreviations

SSR: Simple sequence repeat
PPI: Protein-protein interaction
NPNS: Nonpathogenic *Neisseria* strains
rRNA: Ribosomal RNA
tRNA: Transfer RNA
ORFs: Open reading frames
MCL: Markov clustering algorithm
NJ: Neighbour joining
ML: Maximum likelihood
ATNS: All of the tested *Neisseria* strains
NMS: *N. meningitidis* strains
NGS: *N. gonorrhoeae strains*
COGs: Clusters of Orthologous Groups
VFDB: Virulence factor database
URHP: Unique regions of *H. pylori*
DOOR: Database for prokaryotic operons
eggNOG: Evolutionary genealogy of genes: nonsupervised orthologous groups
UGNP: Unique genes for *Neisseria* pathogens
UGNMS: Unique genes for NMS
UGNGS: Unique genes for NGS.

## Supplementary Materials

Supplementary 1 Figure S1: protein-protein interaction of UGNP and UGNMS.

Supplementary 2 Table S1: the ratio of homologous between genomes.

Supplementary 3 Table S2: (A) UGNP in N. gonorrhoeae 32867; (B) UGNGS in N. gonorrhoeae 32867.

Supplementary 4 Table S3: (A) UGNP in N. meningitidis MC58; (B) UGNMS in N. meningitidis MC58.
