# Supplementary material for: Genus-Wide Comparative Genomics Analysis of Neisseria to Identify New Genes Associated with Pathogenicity and Niche Adaptation of Neisseria Pathogens
Source: Int J Genomics. 2019 Jan 15;2019:6015730. doi: 10.1155/2019/6015730 (PMC6350579; doi:10.1155/2019/6015730)
Supplement: Supplementary 1 — Figure S1: protein-protein interaction of UGNP and UGNMS. [file 6015730.f1.docx]

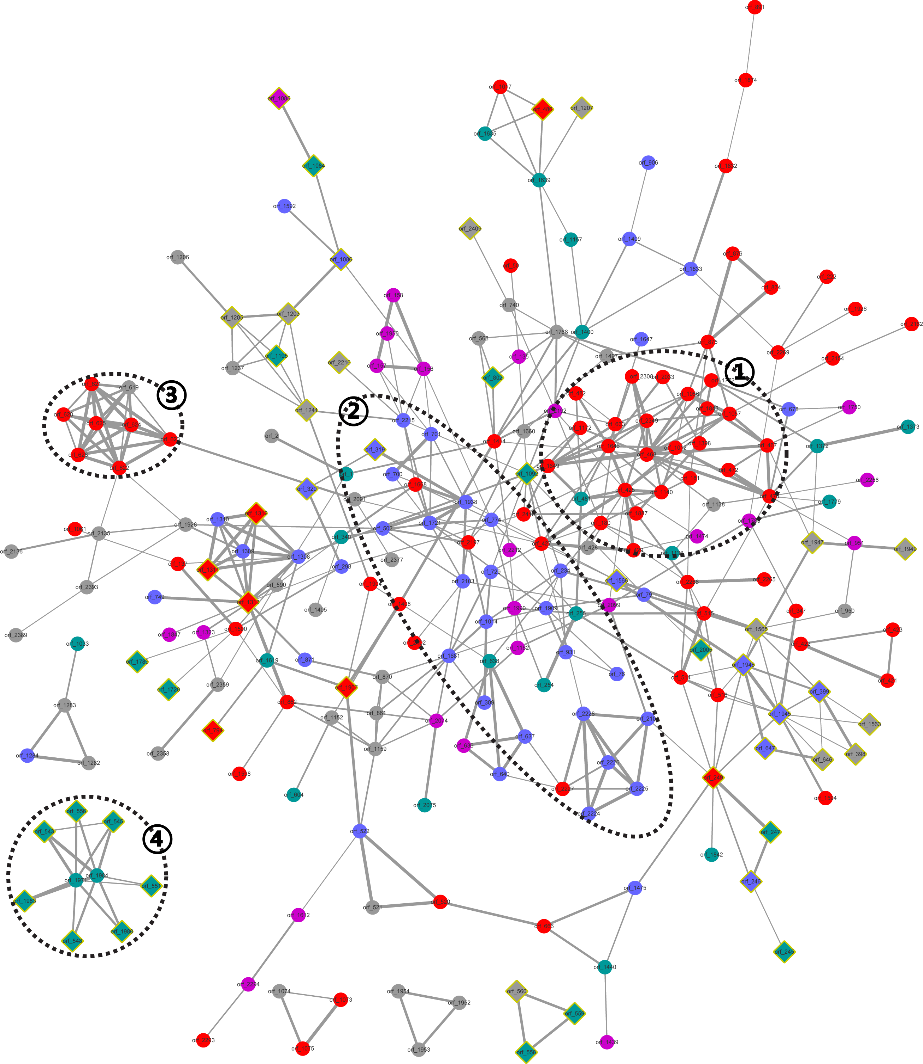


Fig. S1 Protein-protein interaction of UGNP and UGNMS. *N. meningitidis* MC58 genome is used as reference. The names of nodes correspond to Table S2. Circular nodes represent the UGNP proteins and diamond nodes with yellow margin represent the UGNMS proteins. Only the networks that the number of nodes was greater than 3 were shown. The network edges represent the protein-protein associations and the line thickness of them indicates the confidence of the association between corresponding nodes. The disconnected nodes are hided in the map. Different colors represents different protein function categories: red, basic substances transport and metabolism; purple, genetic information processing, including replication, transcription and translation; blue, cellular processes, including cell wall/membrane/envelope biogenesis, cell motility and so on; green, bacteria-environment interaction, including signal transduction, extracellular structures, defense mechanism and so on; grey, function unknown.
